# DEVELOPING A CULTURALLY TAILORED INTERVENTION FOR AFRICAN AMERICAN PARENT–ADULT DAUGHTER DEMENTIA DYADS

**DOI:** 10.1093/geroni/igae098.1865

**Published:** 2024-12-31

**Authors:** Kalisha Bonds Johnson, Karen Lyons, Joan Monin, Gaea Daniel, Wizdom Powell, Kenneth Hepburn

**Affiliations:** Emory University, Atlanta, Georgia, United States; Boston College, Chestnut Hill, Massachusetts, United States; Yale School of Public Health, New Haven, Connecticut, United States; Emory University, Atlanta, Georgia, United States; Headspace Health, Santa Monica, California, United States; Emory University, Atlanta, Georgia, United States

## Abstract

African American older adults are suggested to be two times more likely to develop Alzheimer’s disease and related dementias (ADRD) compared to non-Hispanic White older adults. Thus, there is a need for more African American care partners. The largest group of care partners of African American persons living with ADRD, African American adult daughters, must navigate formal care decision making through the lens of race, gender, culture, and associated health disparities. Formal care—paid health care services for older adults usually associated with an institution— is important in the treatment of ADRD and maintenance of quality of life. Guided by the NIA Health Disparities Research Framework, Black Family Social-Ecological Context Model, and Superwoman Schema (SWS), this presentation will describe the first aim of this explanatory sequential design of a culturally tailored prototype intervention to support the formal care decision-making process for African American parent-adult daughter dementia dyads. Results from the preliminary analysis of 20 dyads highlight most are mother-daughter dyads (90%) with parents, on average, experiencing moderate cognitive impairment. Both parents and adult daughters report “good” health, “some” difficulty with family listening to their opinions, moderate to high decision self-efficacy, and moderate quality of life. Daughters report low SWS characteristics but high cultural justification for caregiving. Using these results, we will showcase the next steps of data collection, how theory guided our study, the intentionality of relationships status, and the role of community engagement as important considerations for dyadic prototype intervention development.

